# Supplementary material for: Multivalent Tau/PSD-95 interactions arrest in vitro condensates and clusters mimicking the postsynaptic density
Source: Nat Commun. 2023 Oct 27;14:6839. doi: 10.1038/s41467-023-42295-2 (PMC10611757; doi:10.1038/s41467-023-42295-2)

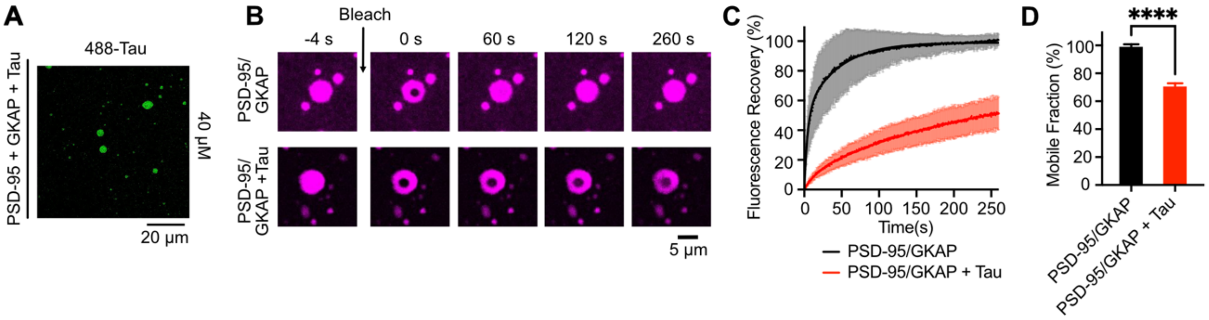

Supplementary Fig. 1 | Tau reduces PSD-95 dynamics in PSD-95/GKAP condensate. A. Confocal microscopy images demonstrating partitioning of Tau into PSD-95/GKAP droplets. Tau was labeled with Alexa 488. The concentration of each protein was 40 µM. **B**. Representative FRAP images of PSD-95 in PSD-95/GKAP droplets without and with Tau. The concentration of each protein was 40 µM. PSD-95 was labeled with Alexa 647. **C**. Quantification of PSD-95 dynamics in FRAP measurements shown in (B). FRAP curves of "PSD-95/GKAP" and "PSD-95/GKAP/Tau" were averaged from 4 and 5 FRAP measurements, respectively (solid lines: fitted FRAP). Error bars indicate standard deviation. **D.** Mobile fractions of PSD-95 derived from fitting the FRAP data displayed in (C). Error bars represent standard deviation of curve fits; unpaired and two-tailed t-test with Welch's correction: ****$p \leq 0.0001$. Source data are provided as a source data file.

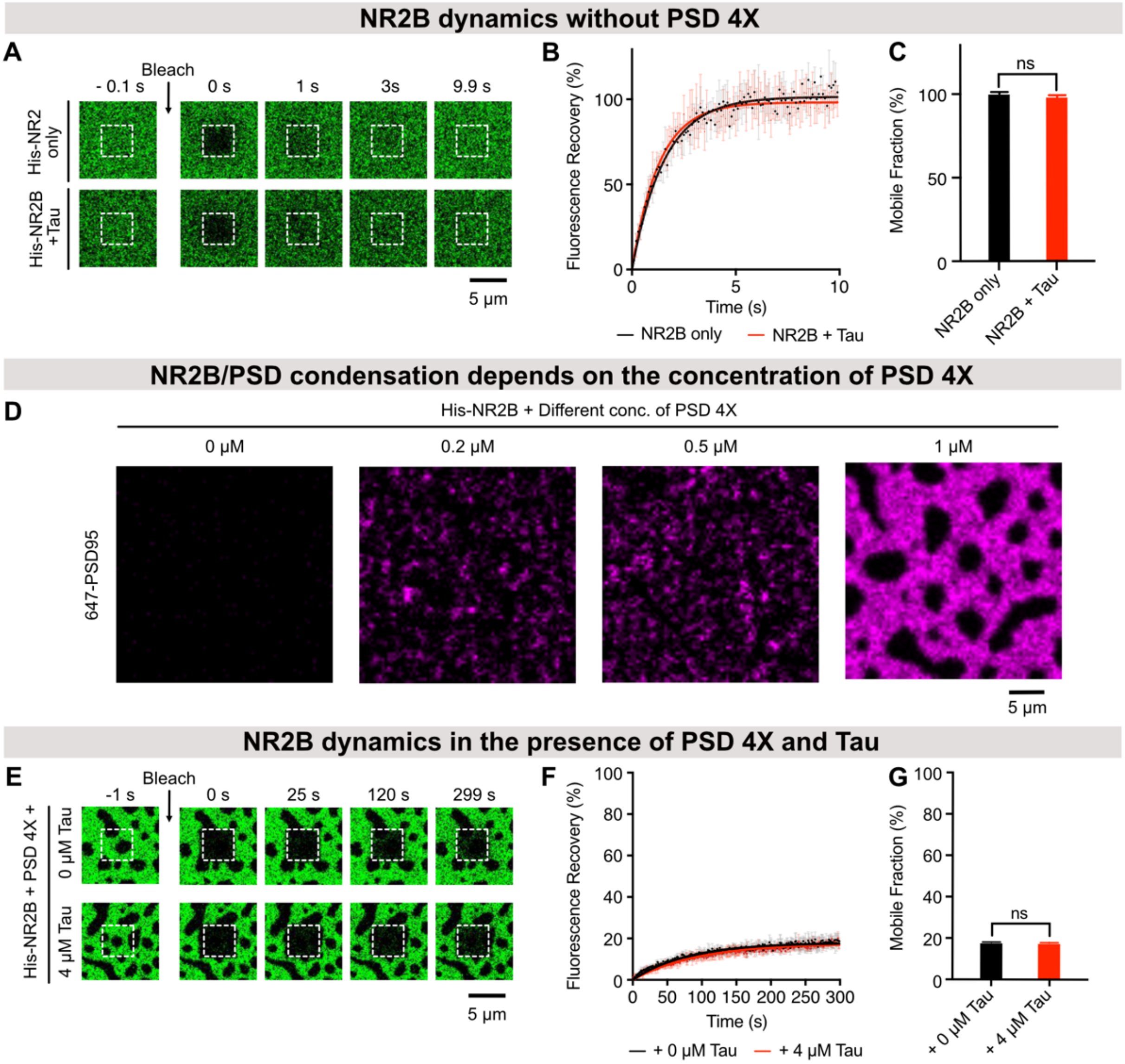

Supplementary Fig. 2 ⎸ Formation and dynamics of membrane-anchored NR2B/PSD condensates. A. Representative FRAP images demonstrating rapid diffusion of His-NR2B in the His-NR2B-only system (top) and the His-NR2B/Tau system (bottom) within 10 seconds. The concentration of Tau was 2.5 μM. His-NR2B was labeled with Alexa 488. **B.** Quantification of the mobility of His-NR2B with and without Tau (from A); averaged from three FRAP measurements and fitted with a mono-exponential function. Error bars indicate standard deviation. **C.** Mobile fractions of His-NR2B derived from fitting the FRAP data displayed in (B). Error bars represent standard deviation of curve fits. Unpaired and two-tailed t-test with Welch's correction were performed. **D.** NR2B/PSD condensation on the membrane depends on bulk concentrations of PSD scaffold proteins.

The four scaffold proteins were mixed at a 1:1:1:1 ratio at the indicated concentration. Representative images were acquired 15 minutes after adding the PSD scaffold proteins. Three measurements were done for each condition. **E.** Representative FRAP images showing His-NR2B dynamics in NR2B/PSD condensates without and with 4 μM Tau. **F.** Quantification of His-NR2B dynamics in the NR2B/condensates shown in (E); averaged from three FRAP measurements and fitted with a mono-exponential function. Error bars indicate standard deviation. **G.** Mobile fractions of His-NR2B derived from fitting the FRAP data displayed in (F). Error bars represent standard deviation of curve fits. Unpaired and two-tailed t-test with Welch's correction were done. Source data are provided as a source data file.

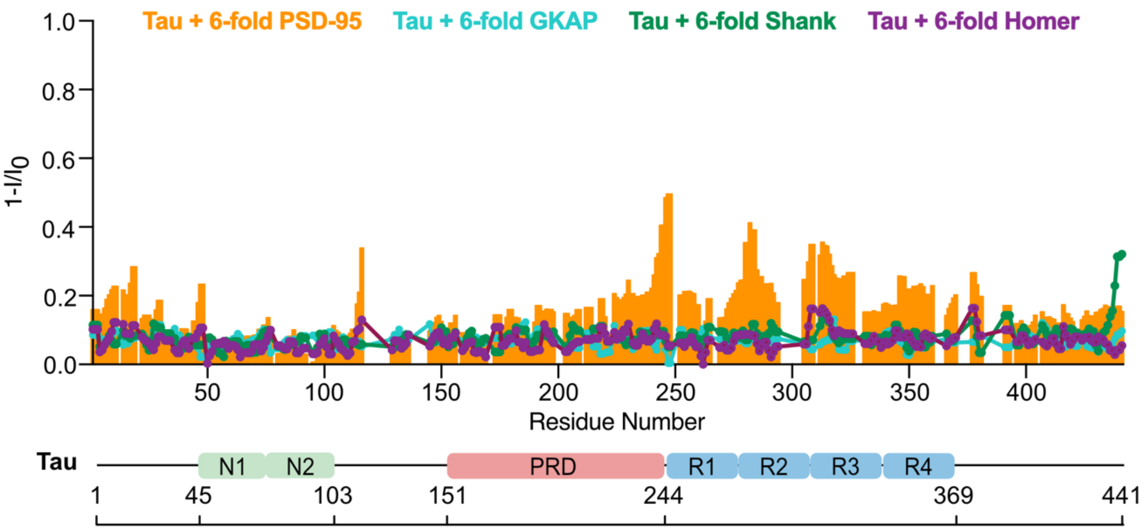

Supplementary Fig. 3 | Interactions between Tau and PSD proteins. NMR signal intensity profiles of Tau upon the addition of 6-fold molar excess of PSD-95 (orange bar), GKAP (cyan lines), Shank (green lines) or Homer (purple lines), respectively. I_0 represents the signal intensities of Tau alone. The domain organization of Tau is shown below the intensity profiles. For Tau/GKAP experiments, 20 µM Tau and 120 µM GKAP were used. For others, 10 µM Tau and 60 µM of PSD-95/Shank/Homer were used due to limited protein stocks. Source data are provided as a source data file.

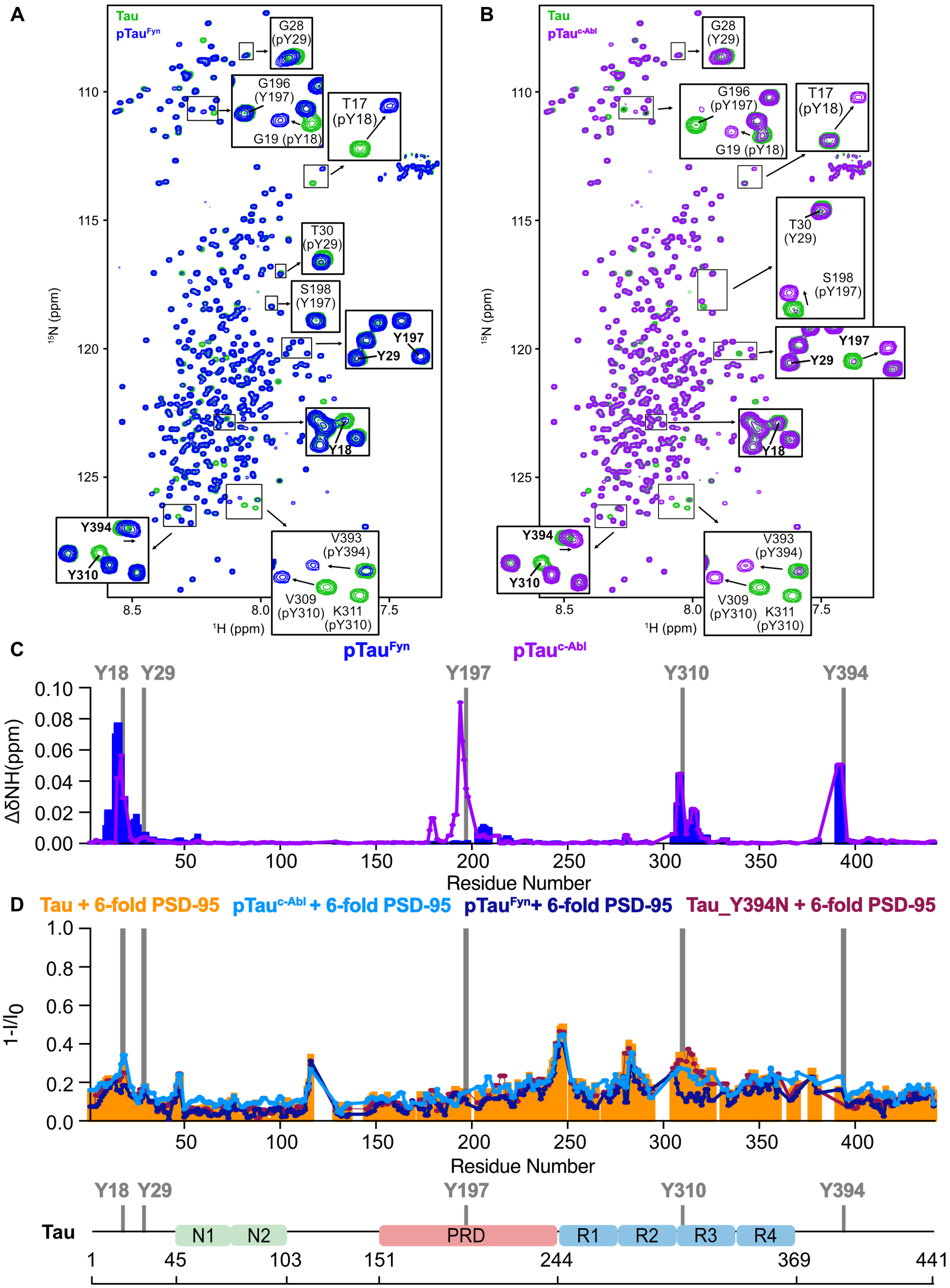

Supplementary Fig. 4 | Tyrosine phosphorylation of Tau and its impact on NMR intensity profiles. A, B. Superposition of ^{1}H-^{15}N HSQC spectra of unmodified Tau (green) with Fyn-

phosphorylated Tau (blue; $pTau^{Fyn}$) in (A) and c-Abl-phosphorylated Tau (purple; $pTau^{c\text{-}Abl}$) in (B). The tyrosine residues being phosphorylated (highlighted in bold) and their neighboring residues are shown in the zoom-in views. **C.** NMR chemical shift perturbation analysis of Tau upon Fyn-mediated (blue bars) or c-Abl-mediated (purple lines) phosphorylation. **D.** NMR signal intensity profiles showing the signal intensity changes of Tau (orange bars), $pTau^{c\text{-}Abl}$ (blue lines), $pTau^{Fyn}$ (dark blue lines), and Tau_Y394N (dark red lines) peaks upon the addition of 6-fold molar excess of PSD-95, with I_0 being signal intensities of Tau alone, $pTau^{c\text{-}Abl}$ alone, $pTau^{Fyn}$ alone and Tau_Y394N alone, respectively. 10 μM of different Tau constructs and 60 μM of PSD-95 were used. The domain organization of Tau is illustrated below the intensity plots. In **C** and **D**, grey bars represent the position of Tau's five tyrosine residues. Source data are provided as a source data file.

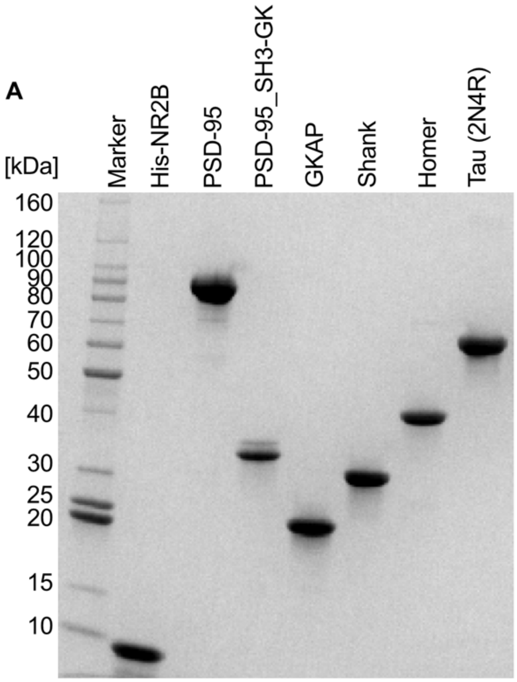

B

Name	Estimated molecular weight (kDa)
His-NR2B	9.0
PSD-95	80.5
PSD-95_SH3-GK	35.3
GKAP	19.2
Shank	30.2
Homer	40.0
Tau	45.8

Supplementary Fig. 5 | Gel-based analysis of purified proteins. A. 2 µg of each protein was loaded into the well of 4%-20% SDS-polyacrylamide gel. **B.** Molecular weights of the proteins calculated by the online Protparam tool (https://web.expasy.org/protparam/). His-NR2B stands for His_6-tagged GCN4-NR2B; PSD-95 and Homer stand for full-length PSD-95 and Homer, respectively; GKAP stands for the GKAP_GBR_PBM construct; Shank stands for Shank_M1718E_PDZ_HBS_CBS_SAM construct; Tau stands for full-length human 2N4R Tau.

Scanned Gel Image Related to Supplementary Fig. 5 A

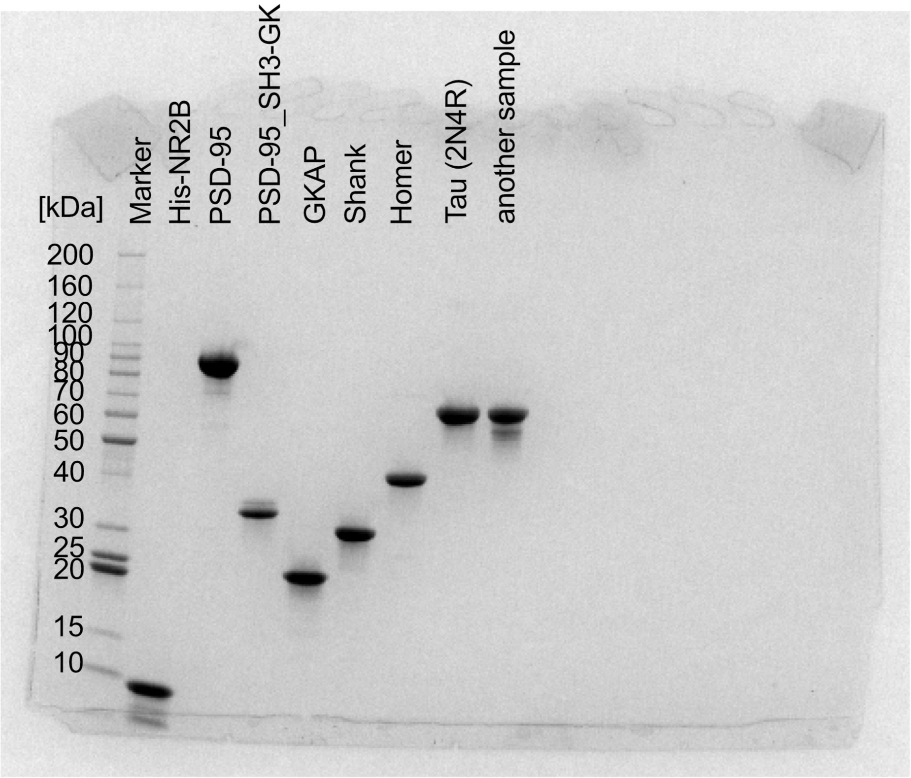

Supplement: Supplementary file 1 — Supplementary Information [file 41467_2023_42295_MOESM1_ESM.pdf]
